# Variabilities of gallbladder contraction indices and a simple regression model for gallbladder and gastric emptying ratio

**DOI:** 10.4314/pamj.v9i1.71186

**Published:** 2011-05-31

**Authors:** Ugwu Anthony Chukwuka, Agwu Kenneth Kalu, Okechukwu Felix Erondu

**Affiliations:** 1 Department of Radiography and Radiological sciences Nnamdi Azikiwe University, Nnewi Campus, Anambra State, Nigeria; 2 Department of medical radiography and Radiological sciences, University of Nigeria , Enugu campus, Enugu state, Nigeria; 3 Department of clinical imaging, Image Diagnostics, Port Harcourt, Rivers State Nigeria

**Keywords:** Gallbladder, contraction indices, gastric empty ratio, variabilities

## Abstract

**Introduction:**

The objective of this study was to assess the variabilities of gallbladder contraction indices (GBCI) and derive a predictive model for gallbladder and gastric motility.

**Methods:**

The gallbladder volume and gastric antral measurements were obtained from 24 healthy male volunteers in preprandial and post-milk ingestion states. After preprandial measurement of the gallbladder volume and gastric antral area, each subject ingested 157 ml of full cream milk and 30 cl of ion -free water. In supine position, the gallbladder volume and the gastric antral area were obtained every five minutes for 40 minutes. For the gallbladder while only the 5 ^th^, 10 ^th^ and 15 ^th^ measurement of gastric antral area were obtained. Gallbladder contraction indices were calculated and gastric emptying ratio obtained at the fifteenth minute is the indication of gastric motility. Statistical analyses were conducted using SPSS version 16.0 with p<0.05 as criterion of statistical significance.

**Results:**

The GBCIs followed Gaussian response at some stages and did not at some other stages. The least variability occurred at the 35th measurement of GBCI. A cut- off value for the 35th minute GBCI value was established with the mean±2 SD (80.79±11.5%). Obvious gallbladder refilling was noted after 35minutes. A positive relationship was noted between gallbladder and gastric motilities.

**Conclusion:**

With milk dilution, the variability of gall bladder motility is least at the 35th minute. A significant positive relationship between gastric emptying and gallbladder contraction index was also observed.

## Introduction

Impairment of gallbladder emptying is increasingly suspected to be a potential pathophysiological factor in the development of gallstones [1,2]. Previous studies described the influence of sex hormones on gallbladder emptying.

A significant delay in gallbladder emptying during the luteal phase of the menstrual cycle and increased incidences of cholelithiasis in women using sex hormones have been reported [3,4]. Postprandial gallbladder motility has been found to be reduced in obese compared to non- obese women [5]. This difference could be attributed to higher incidence of gallstones in obese subjects. The mathematical analysis of minute-by-minute ultrasonography measurements of gallbladder volume variations yields both physiological and pathological insights. In healthy volunteers it has been shown that the fat content of meals affects the mode of gallbladder emptying and refilling [6]. Both cholecystokinin (CCK) infusion and milk ( fatty meal) ingestion have been used to induce gallbladder contraction in cholecystodynamic studies.

The commonest examples are CCK-cholescintigraphgy and milk ultrasonography. In 2001, Ziessman and co-authors reported that a 3-minute infusion of sincalide (0.01 µg/kg), produces too variable a gallbladder ejection fraction (GBEF) response to establish a useful normal range. With a 0.01 µg/kg, diluted in a 30 ml volume, infused for 60 minutes, clinical useful normal values were established at 40 and 60 minutes [7]. A previous study utilized undiluted full cream milk and assessed gallbladder motility for just 20minutes. The variabilities of gallbladder contraction indices (GBCIs) at different periods was, however not taken into account in this study [8]. How variations in this factor and longer periods would affect gall bladder motility are yet to be investigated. This study was designed to use a milk dilution method to assess the variabilities of gallbladder motilities at different periods until an obvious gallbladder refilling occurs. Due to vagal motivation of gastric and gallbladder motilities, this study also established a predictive regression model for gastric and gallbladder motilities. To the best of our knowledge, this approach has scarcely been explored.

## Methods

This study was approved by the Human Research Ethnics Committee of St Charles Borrome Hospital, Onitsha, Anambra State. Signed informed consent was provided by 24 consecutively enrolled male volunteers who met the criteria for inclusion. All the subjects were in good health. Detailed medical histories were obtained to exclude any subject with symptoms of recurrent abdominal vomiting, or a medical history of hepatobiliary disease. Other exclusion criteria include positive history of diabetes, achalasia, irritable bowel syndrome, truncal vagotomy, pancreatic insufficiency, sickle cell hemoglobinopathy, and hemolytic anemia. Subjects who have received any form of medication at least 10 days before the study were also excluded.

After an overnight fast, the subjects arrived at the department between 0700hours and 0900 hours. The pre-prandial measurements of the gallbladder and gastric antrum were obtained using ultrasonography. The length, width and antero- posterior measurements of the gallbladder were obtained. The basal (pre-prandial) longitudinal and anteroposterior dimensions of the gastric antrum were obtained using the left lobe of the liver and the abdominal aorta as internal landmarks [9]. The methods for obtaining gallbladder measurements have been previously described [8]. Measurement were obtained using a 3.5 MHz curve linear transducer ( Siemens Sonoline SL-2, Issaquah, USA) After pre-prandial measurements (in supine position) each subject ingested a tin of full cream Peak brand milk (157ml, 170g, contents, vitamins and iodine. Milk fat 9%, milk solids not fat 22%, milk stabilizer E339m, brand of Friesland foods, WAMCO Nigeria Plc). This was immediately followed by ingestion of 30 cl of ion -free water (Eva water, Coca Kola Co, Plc). This gave a 457 ml of liquid meal (milk and water). One minute was allowed for both milk and water intake. The combination of water and milk is to make the liquid meal palatable to the participants, induce gallbladder contractility and create enough volume that can be assessed sonographically. The gallbladder and gastric antral measurements were taken every five minutes for forty minutes while the gastric antral measurements were obtained at fifth, tenth and the fifteen minutes. The gallbladder volumes were assessed by the ellipsoid method [10]. The gastric antral area was assessed using a standard formula [9]

Gallbladder contraction index was calculated as the percentage change in volume at each period using the fasting volume as baseline volume.

The gastric emptying rate was calculated as the gastric emptying ratio of basal antral area to gastric antral area at the 15th minute post prandial [11]. At the end of the procedure, subjects heights were measured on a calibrated vertical wall and the weight measured on a weighing scale (Model H 89 LT Blue). The subject′s age was also obtained. The body mass index (BMI) was measured in kg/m^2^ while the body surface area (BSA) was calculated using a standard equation [12]. Statistical analyses were conducted using SPSS software version 16.0 (SPSS Inc, Chicago, Illinois, USA) and graph drawn on Microsoft Excel. Gaussian responses of GBCI values were conducted using Kolmogorov- Smirnoff test. Both inferential and descriptive statistics were applied to the data. P< 0.05 was used as a criterion of statistical significance.

## Results

Ages of the participants ranged from 27 years to 40 years with a mean±standard deviation of 33.75±4.12 years. The weight, height, body surface area( BSA) and body mass index ( BMI) were 55kg- 69kg ( 65±5.96kg); 1.62m- 1.76m ( 1.68±0.06m); 1.58m^2^-1.82 m^2^ ( 1.74+0.1 m^2^) and 20.7 kg/m^2^ - 26.29 kg/m^2^ (23.2+ 2.21kg/m^2^) respectively.

### Gallbladder contraction indices and gastric emptying ratio

**Figure 1:**
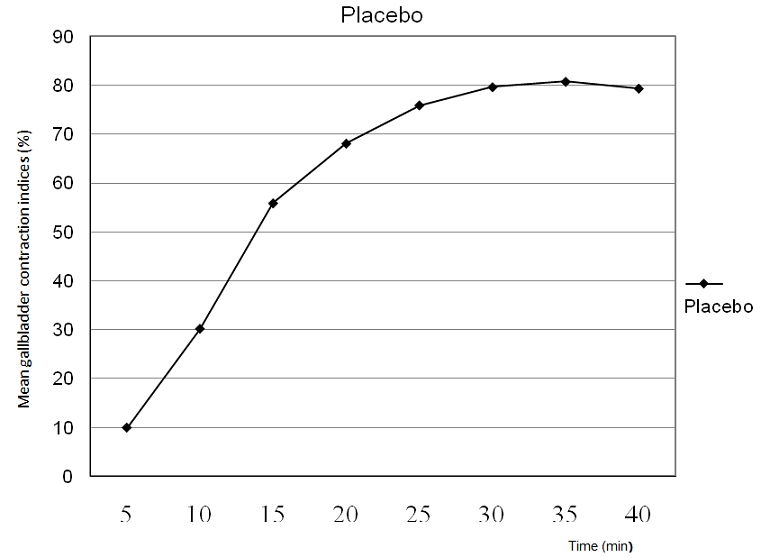
Diagram showing the gallbladder contraction curve

The mean GBCIs and their coefficients of variation were 9.94% (158.5%), 30. 2% (54.3%), 55.88%( 15.9%), 68.12%( 12.3%), 75.96% (11.2%), 79.68%( 9.86%0, 80.79% (7.1%) and 79.4% (12.75%) for the 5th, 10th, 15th, 20th, 25th, 30th, 35th and 40th minutes respectively. The gallbladder motility curve is shown as Figure 1. The mean value of the sum of the 10th and 20th minute GBCIs was obtained to be 48.78%( GBCIu). The 15th minute gastric emptying ratio (GERA) was 1.56±0.2 (mean±SD). The mean gastric antral area after ingestion of liquid meal was noted to be 1.1 cm^3^, 0.7 cm^3^ and 0.6 cm^3^ at the 5th, 10th and 15th minute.

Obvious gallbladder refilling was noted after the 35^th^ minute as shown in the gallbladder contraction curve (Fig. 1). The maximum contraction difference between a GBCI value and a succeeding GBCI value occurred between the 5th and 10th minute GBCI (30.2-9.94=20.26%). Kolmogorov- Smirnoff test indicates that GBCIs at 5^th^, 10^th^, 20^th^ and 30^th^ minute showed non Gaussian responses. The rest GBCI values followed Gaussian (normal distribution) responses. The least coefficient of variation ( C of V) for GBCIs was found with GBCl 35, hence GBCl 35 was adopted as the GBCl for this study upon which clinical cut- off values were established using a 2 sigma rule and a 5% level of significance ( type 1 error). A mean GBCI35±2SD (Standard deviation) was established thus 80.79±11.50%. GBCI35 showed no significant correlation with BSA, age and weight but significantly correlated with BMI (r=0.81, P =0.00), and Height (r=0.83, p= 0.00) A multiple linear regression (predictive) equation for GBCI35 is derived thus: GBCI35=61.6 height (m)-1.53 BMI (kg/m^2^)+12.87. Gallbladder refilling and possible cessation of CCK release were made evident by the closeness of the GBCI values at the 30th, 35th, and the 40th minute, while the maximal contraction difference in the 5th to 10th minute contraction, reduced contraction and obvious refilling (Fig. 1) represents the three phases of gallbladder motility: contraction phase, tonic phase and refilling phase. Pearson′s correlation between GBCI35 and GBCI40 (indirect measure of refilling) showed an insignificant relationship (r=0.38, p= 0.07). This indicates that the rate of gallbladder contraction and refilling are not related. Lower GBCI values at the 40th minute (refilling period) indicate faster gallbladder refilling. GBCI40 did not significantly correlate with any of the body indices (weight, height, BMI, and BSA). The GBCI40 did not significantly correlate with GERA15 indicating no relationship between gastric emptying and gallbladder refilling. GBCI35 showed a negative and significant correlation (r= -0.58, p=0.03) with GERA 15, indicating an increase in gastric motility as gallbladder motility increases. A simple linear regression (predictive) equation for GERA15 and GBCI35 is derived thus:

GERA15=-0.01GBCI35 (%)+2.38.

Low values of GERA15 indicate higher gastric emptying rate.

## Discussion

The formation of cholesterol supersaturated bile in patients with cholesterol gallstone disease is causatively related to decreased gallbladder contractility [13]. Both CCK-infusion and milk (fatty meal) ingestion have been used in cholecystodynamic studies. The gold standard for assessing gastric and gallbladder motility is dynamic scintigraphy. Ultrasonography is also a valid method for these studies but has the advantage of being safe, cheap, non invasive and does not make use of ionizing radiation. Some previous studies used CCK- infusion to induce contraction of the gallbladder [7,14]

It has also noted that the use of CCK does not offer any advantage over the ingestion of fatty meals in radiographic studies involving induced contractions [15]. At present, literature has not shown any generally recognized parameter for quantification of gallbladder motility. Neither spontaneous alterations of gallbladder size nor the variability of repeated measures have been considered or standardized. This makes it difficult to compare contradictory reports in literature [7,16]. The results of this study show that the GBCI values at some periods followed Gaussian response (normal distribution) and at some others were non Gaussian. This partially agrees with the report by Krishnamurthy and Krishnamurthy which noted that the gallbladder ejection fraction is non- Gaussian [16]. The least variable GBCI was obtained at the 35th minute. Variability at different periods was not the same possibly due to blood concentration of CCK and pancreatic polypeptide both of which impact on gallbladder motility at different periods.

Obvious evidence of gallbladder refilling was also observed at the 40th minute postprandial (Figure 1). The mean±2SD for GBCI35 was obtained with a 5% false positive (type 1 or alpha) error. In line with Bayes theorem, this 5% level of significance (type 1 error) will decrease in epidemiological sample as patients with high pretest likelihood have high chances of true positive results, having undergone extensive clinical work up prior to referral.

Significant correlations between GBCI35 and BMI and between GBCI35 and height were noted in this study. This partly agrees with the report of a previous study which indicated that GBCI correlated significantly with height and insignificantly with BMI [17]. The multiple linear regression model derived in this study for GBCI35 would be useful in the prediction of GBCI prior to or after milk cholecyto-sonograpy in gallbladder diseases or high risk patients.

This study also shows a negative but significant correlation between GERA15 and GBCI35. As higher GERA values translate to reduced gastric emptying, this negative relationship is in agreement with the normal physiological processes, as both gastric and gallbladder motilities are autonomically controlled by the Vagus nerve. The regression equation for GERA15 and GBCI35 could also be useful in predicting any suspicion of poor function, in clinically normal subjects. In healthy volunteers, it has been shown that the caloric content of meals affect the total amount of bile recycled by the gallbladder; and that the fat content of meals affects the rate of gallbladder emptying and refilling [6]. In a previous study which adopted the use of an undiluted tin of milk (higher concentration of fat), the mean values at the 10th and 20th minute GBCI±SD was 42.8±19.33 giving a 45.16% coefficient of variation [8]. In the present study which allowed for milk dilution with 35 cl of water, the equivalent value (GBCIu) of 48.78±5.56% with a coefficient of variation of 11.41% was obtained.

Less variation with the present study indicates that better clinically useful values could be obtained with milk dilution. The wide difference in variability in these two studies gives credence to the finding that fat content of meals affect the modality of gallbladder emptying [6]. Though, the cumulative fat content was similar, the fat concentration per unit volume of the liquid meal was higher in the previous study where dilution was not adopted [17].

Unlike the left ventricle which has a fixed ejection fraction at a given time, gallbladder ejection fraction (GBCI) can be varied by changing the fat content of the meal. Higher concentration of fat in the meal ought to shift the mean GBCI to the right. This was not observed between the present and previous study possibly due to the anthropometric characteristics of subjects evaluated in the two studies [17]. The GBCI 35 in this study did not correlate significantly with age.

This finding agrees with those of Wedmann et al [17]. Similar to a previous study [18], no significant correlation between gallbladder contraction index (GBCI35) and gallbladder refilling (GBCI40) was noted in this study. GBCI40 also did not show any significant correlation with any of the anthropometric variable. Faster refilling means the sphincter of Oddi regains its tone faster, thus facilitating refilling of the gallbladder. The fraction of hepatic bile that enters the gallbladder is controlled, principally by the tone of the sphincter of Oddi. Noticeable gallbladder refilling was observed at the 40th minutes post prandial assessment. It is important to know how soon after ingestion of fatty meal or infusion of CCK, the gallbladder begins to fill again. If it does not fill early enough, a normal gallbladder might suggest cystic duct obstruction of even poor hepatic function.

Maximum difference between a GBCI value and a succeeding GBCI value in this study was noted between the 5th and the 10th minute GBCIs (approximately 21%). This observation showed a relationship between this maximum contraction and the maximal gastric antral cross sectional area which occurred at the 5th minute. The above finding suggests a maximum influx of liquid meal from the antrum into the duodenum between the 5^th^ and the 10^th^ minute, the time at which the maximum contraction difference occurred, indicating greater motility. CCK is released endogenously by the duodenal mucosa in response to a fatty meal.

The serum CCK rises and peaks during 20-30 minutes and remains elevated until there is no longer a stimulus for secretion; after the meal has passed through the upper small bowel. The serum CCK then promptly returns to baseline due to its rapid metabolism (2.5 minutes serum half-time) [19]. The occurrence of a maximum gallbladder contraction difference of 21% (GBCI10-GBCI 5) and a decline afterwards is in one accord with this reported 2.5 minute serum half-life of serum CCK. With a longer half life, it would be expected that this contraction difference would have maintained a plateau between the 10^th^-15^th^ (15%) and the 15^th^-20^th^ (13%) minutes GBCI values. Gallbladder contraction is initiated when the serum CCK reaches a threshold that is considerably lower than peak CCK [19]. Simultaneously, CCK relaxes the sphincter of Oddi, allowing bile to empty into the small bowed where it facilitates fat absorption. The maximum contractility between the 5th and the 10th minute and the corresponding maximum gastric antral area in the 5th minute indicates a maximum inflow of fatty meal into the duodenum between the 5th and the 10th minute, giving rise to this maximum contractility. This result indicates a possible maximum CCK release from the duodenal mucusa between the 5th and 10th minute in this study contrary to the 20-30th minutes reported other authors [19].

As these two studies were conducted at different periods and with different meals, this finding is also giving credence to the report that the fat content of meals affects the modality of gallbladder contraction and refilling [6]. The impact of fat content of meal on gallbladder refilling was also noted in a previous study which showed obvious gallbladder refilling after 60 minutes [20]. However the present study which showed refilling of gallbladder after 35 minutes. Nadir of GBCI differences were obtained at the 25^th^, 30^th^ and 35^th^ minute indicating a possible near cassation of CCK release at there periods. The 30^th^ and 35^th^ minutes GBCI values were noted to be less variable than the earlier values of GBCI in this study.

The decrease in variability as timing increases and the decrease in variability with milk dilution observed in this study and compared to a previous study, [8] are congruent to the study by Ziessman et al [7]. This showed that a 3- minute infusion of sincalide, 0.01µg/kg, produces too variable a gallbladder ejection fraction (GBEF) response to establish a useful normal range. With 0.01µg/kg infused for 60 minutes, clinically useful normal values were established at 45 and 60 minutes. The normal values, mean±2SD obtained in these studies have a 5% false positive (type 1) error when validated on healthy subjects. This deduction is in agreement with that of the study just described, which triggered a counterpoint by Krishnamurthy and Krishnamurthy [16]. The latter argued that error limit (rates) can only be established when established values are tested on epidemiological samples (patients with a pretest probability of suffering from the particular diseases), which the earlier did not do [7]. This submission is wrong and correct. It is wrong because normal values can be established and validated in healthy subject in the same population/tribe or in a different population.

In this situation, error limits for healthy subjects that would be adjudged to have the diseases condition would be known. This is called false positive error (type 1) or alpha error. The submission is correct as validating the normogram (normal values) on epidemiological sample would give an error limit [16]. This error limit would be those that have the diseases and the normogram would adjudge them to be diseases free. This error limit or rate is called false negative rate (type 2) or beta error.

The major limitation of this study is that dynamic cholecystosonography unlike dynamic cholescintigraphy does not record or account for inflow of bile during the calculation of GBCI. As the gallbladder contracts following ingestion of a fatty meal (to release bile), inflow of bile into the gallbladder also occurs. This becomes prominent when CCK release ceases and obvious refilling occurs. With dynamic cholescintigraphy ejection is recorded without interference on its values by inflow of bile. Future study in this area; adopting the use of dynamic cholescintigraphy, is recommended.

## Conclusion

This study has shown variablities of GBCI values at different periods and indicates that milk dilution and obtaining values at peak periods (periods close to obvious refilling time) are better ways of establishing clinically useful cut- off values. It can therefore be employed as a predictive model for gallbladder and gastric motility and provide useful guides to clinical decisions in patients with gallbladder disease. A significant positive relationship between gastric emptying and gallbladder contraction index was also observed.
